# Searching for Meaning in the Life of Ellen Johnson-Sirleaf: A Call to Meaningful Responses to Tragedies

**DOI:** 10.17505/jpor.2023.25815

**Published:** 2023-12-07

**Authors:** Tinashe Timothy Harry, Roelf van Niekerk

**Affiliations:** Department of Industrial and Organisational Psychology, Nelson Mandela University, Port Elisabeth, South Africa

**Keywords:** Ellen Johnson-Sirleaf, non-WEIRD, psychobiography, Viktor Frankl, leadership, logotherapy

## Abstract

Ellen Johnson-Sirleaf, born in 1938, is Africa's first elected female head of state. She navigated stressful and traumatic events, including bullying, domestic abuse, persecution, a civil war, witnessing the effects of genocide and navigating a patriarchal system. Nonetheless, Johnson-Sirleaf's determination established her as a global icon who played a significant role in women’s empowerment. Johnson-Sirleaf was purposively selected for this study as she is an extraordinary female political leader. Frankl’s dimensional ontology is employed to describe and explore the life of Johnson-Sirleaf from a psychobiographical perspective using publicly available information. The study focused on the period between 1938 and 2005. The findings suggest that Johnson-Sirleaf transcended psychophysical and psychosocial limitations and crafted a meaningful life. It seems Johnson-Sirleaf was guided by a sense of purpose and used her will to meaning to overcome psychosocial injustices and psychophysical issues. This study illustrates the value of Frankl’s existential theory in illuminating the life histories of extraordinary political leaders and its potential contribution to contemporary mental health challenges.

## Introduction

There have been calls for psychobiographical studies focusing on extraordinary women in diverse contexts (Mayer, 2022, 2023; Mayer et al., 2022; Panelatti et al., 2021). Ponterotto (2019) argued that psychobiographies allow us to develop an enhanced appreciation of individuals’ development, values, inner attitude, struggles, challenges, and successes. This study employs a psychobiographical approach to explore, describe, and interpret the search for meaning by Johnson-Sirleaf (born in 1938). The present psychobiography focuses on a period between 1938 to 2005. The researchers selected this period as it is characterized by various transitions in her personal life and career development, culminating in her becoming the president of Liberia in 2006. During this period, Johnson-Sirleaf experienced a range of life-threatening circumstances. More specifically, she was a victim of bullying behaviour and domestic violence and experienced exile, civil war, imprisonment, and navigating a patriarchal society (Berger, 2010; Cooper, 2017; Wilson, 2015).

Johnson-Sirleaf was the first democratically elected African female president, making her one of the most influential women in Africa and the world (Scully, 2016). Johnson-Sirleaf comes from a non-Western, non-Educated, non-Industrialised, non-Rich, and non-Democratic (non-WEIRD) background (Mayer, 2021; Mayer, 2022). Johnson-Sirleaf is one of the leading promoters of democratic rule, women empowerment, peace, justice, and freedom (African Center for Economic Transformation [ACET], 2022; Wellesley College, 2019). She has received numerous accolades, including the Presidential Medal of Freedom (the United States’ highest civilian award) in 2007, the Nobel Prize for Peace in 2011, and the African Gender Award (2011). She has also been described as one of the most powerful women in Africa and one of the 100 most powerful women globally by Forbes (2011; 2012) (Cooper, 2017; Scully, 2016; Wilson, 2015) and the Economic Times (2010) described her as Liberia's best-ever president. She has inspired many individuals through her dedication to women's empowerment, and much can be learned about how she lived a meaningful life in appalling circumstances. Undertaking such a study allowed the researchers to gain a perspective on how Johnson-Sirleaf made sense of her experiences and discovered meaning within her life experiences.

### Aim of the study

This study aimed to investigate how Johnson-Sirleaf pursued meaning within the first 68 years of her life. The aims of the study were four-fold: to (a) describe the challenges that Johnson-Sirleaf faced; (b) interpret events and experiences using Frankl’s dimensional ontology, in particular, the noetic dimension; (c) contribute to research focusing on the role and influence of meaning in psychobiographical research (Mayer et al., 2021a; 2021b; Mullen, 2019); and (d) contribute toward psychobiographical research focusing on women political leaders from non-WEIRD contexts (Mayer, 2021; Mayer, 2022).

### A brief history of Liberia (1989 – 2003)

Liberia is a small West African coastal country founded in 1822 as a settlement area for returning enslaved people (their descendants are generally known as Americo-Liberians) from the United States of America (Cook, 2018; Dennis, 2006). Liberia experienced one of the bloodiest civil conflicts in African history, in the form of two civil wars, the first between 1989-1996 and the second between 1999-2003 (Cook, 2018; Dennis, 2006; Kieh, 2009a, 2009b; Momodu, 2016). The underdevelopment of the country and the poor performance of President Doe's regime are regarded as the leading causes of the first civil war (Kieh, 2008; 2009a, 2009b; 2016). The civil war resulted from a conflict between rebels led by Charles Taylor and the militia that supported President Doe and his government (Momodu, 2016). Liberia held presidential elections in 1996 in which Taylor defeated Johnson-Sirleaf.

The second civil war was caused by failed transitional processes (e.g., disarmament and reintegration of ex-combatants) and the horrendous performance of Taylor’s regime in domains such as the economy and security, unequal distribution of power and wealth (Dennis, 2006; Kieh, 2016). In 2003, Liberia was named one of the world’s worst places, characterised by “warfare, banditry, disease, land mines and violence” (Cooper, 2017, p. 135). The second civil war ended when Taylor resigned in 2003 and went into exile. It is reported that the war claimed the lives of thousands, and millions of others were displaced internally and into neighbouring countries as refugees, and caused widespread traumatisation (Kieh, 2016; Herbert, 2014; Momodu, 2016).

Even before the civil wars, Liberia was characterised by “elite politics, corruption, judicial limbo, political, military and economic violence, generational and other group clashes, and widespread poverty” (Bøås & Utas, 2014). The civil wars exacerbated existing challenges such as inequality (ethnic minorities, gender, class, region, youth), unequal access to employment opportunities, essential services (e.g., health, education, water), and famine (Dennis, 2006; Herbert, 2004; Kieh 2009a, 2009b).

### Women and leadership

Women are generally inhibited from having careers due to patriarchal beliefs and values (traditional systems), which continue to disempower and dispirit women (Christensen, 2019; Oakley, 2022; Mayer, 2023; Mayer, 2022). Africa is generally considered the worst place to be a woman on earth due to the prevalence of harmful traditional values and beliefs (Cooper, 2017; United Nations Human Rights Office of the High Commissioner, nd). Despite significant advances in the continent’s social, economic, and political development, African women are widely marginalised within the corridors of leadership (Amina & Ibrahim, 2019; Chaudhary & Dutt, 2022; Mayer, 2022, 2023). However, most existing research on women and leadership has been in the so-called WEIRD contexts (Chaudhary & Dutt, 2022; Henrich et al., 2010; Mayer & Oosthuizen, 2020). It is therefore important to understand how women in other societies in non-WEIRD contexts encounter and experience stressful and challenging environments.

Many women are still perceived as primary caregivers and supporters of their husbands’ careers (Amina & Ibrahim, 2019; Christensen, 2019). Such perceptions have made it difficult for women to be considered for leadership positions (Amina & Ibrahim, 2019). Women are expected to conform to the patriarchal framework, resulting in them being devalued, distorted, and marginalised in the name of wifehood and motherhood (Mayer, 2022; Oakley, 2021). Although women are regarded as free (in the sense of the word), studies show that women do not pursue leadership roles or challenge the status quo due to fear of repercussions such as being ostracised (Oakley, 2021). In addition, women face challenges such as domestic violence, high rape, and murder rates, and microaggressions (Braun-Lewensohn & Mayer, 2020).

Studying the lives of women leaders can show that their lives are not predetermined, as they still have some freedom to choose and have a role in changing their individual and societal trajectories (Fabry, 2021; Frankl, 2006). Furthermore, throughout history, we have been preoccupied with existential concerns, including meaninglessness, hopelessness, and emptiness (Fabry, 2021; Frankl, 2006; Shantall, 2021). The COVID-19 pandemic has left an indelible mark on this generation and has intensified these existential concerns (Van Tongeren & Van Tongeren, 2021). Our current times are filled with instabilities and uncertainties as we live in a world filled with suffering, tragedy, and despair, which affect how we respond to the inner voice of meaning (Van Tongeren & Van Tongeren, 2021). Yet, simultaneously, it is a world filled with extraordinary and meaningful experiences and moments waiting to be discovered (Shantall, 2020).

### Theoretical lens: Frankl's existential theory of meaning

Frankl’s existential theory covers a wide range of concepts regarded as fundamental in finding meaning. In this study, the main focus is on Frankl’s noological or spiritual dimension, an ontic totality view of humans, to explain the pursuit and discovery of meaning by Johnson-Sirleaf. Frankl’s theory postulates that humans are tri-dimensional, consisting of the somatic (biological or physical body), psychological (psyche) and spiritual (noetic) dimensions (Barnes, 2000; Frankl, 2006, 2014; Lukas & Schönfeld, 2019). It is argued that the spiritual dimension, also referred to as the human dimension or the medicine chest of logotherapy, is the essence of the human condition (Fabry, 2013; Wong, 2016). Frankl accepts that psychophysical and psychosocial factors impact human beings, but within his theory the spiritual self is viewed as the most important factor (Barnes, 2000; Fabry, 2013; Frankl, 2014; Marshall & Marshall, 2012). The spiritual dimension represents the defiant power within humans, allowing for the discovery of meaning through the transcendence of psychosocial and psychophysical factors (Lukas, 2020).

The human spirit is also composed of the spiritual unconscious, which possesses a dynamic quality often referred to as noodynamics, contrasting it with Freud’s instinctual unconscious (Barnes, 2000; Lewis, 2019). Noodynamics represents healthy tension between what a person is and what a person has a vision of becoming (Barnes, 2000; Lewis, 2019; Shantall, 2020). In the spirit, the aim is not equilibrium or the removal of tension but rather dynamic development and growth (Barnes, 2000). Therefore, individuals can engage the spiritual determinants to facilitate the discovery of meaning. The human dimension includes freedom of choice, commitment, self-transcendence, goal orientation, faith, ideas, and ideals (Fabry, 2013, 2021; Lukas, 2020; Lukas & Schönfeld, 2019). The selected spiritual dimension resources relevant to this investigation are outlined in the following section.

**Conscience**. Frankl (2014) defined conscience as “an intuitive capacity to find out, to ‘sniff’ out the unique meaning gestalt inherent in a situation, what is meant in a specific situation” (p. 115). It is regarded as the compass needle that points in the direction of *the meaning of the moment* (Fabry, 2013; Frankl, 2014). Conscience is perceived as the centrepiece of the noetic dimension (Fabry, 2013; Lukas & Schönfeld, 2019; Shantall, 2020). True conscience is represented not only by societal, religious, or parental norms but also by behaviour based on free choice (Fabry, 2013; Lewis, 2019). Fabry (2013) argued that ideally, humans act out of their own volition and not because they are driven or afraid of punishment. The muffled voice of the conscience informs humans of their tasks and, by doing so, directs them to the unique meanings of their lives (Frankl, 1985a, b; 2013; Shantall, 2020).

**Will to meaning**. The primary goal of humans is not to find pleasure or power but the desire to discover the purpose of their lives (Barnes, 2000; Frankl, 2006). If the will to meaning is repressed or ignored, it usually leads to feelings of emptiness or frustration (Fabry, 2013). Frankl argued that power is not an end but a means to find meaning (Barnes, 2000; Frankl, 1985a, b; 2006; 2014). Hence, every human is motivated by longing and striving for meaning. Frankl (2006) postulated that an individual must be able to make sense of their lives and life circumstances to satisfy the will for meaning. There has been considerable empirical support for the claim that the will to meaning can be an antidote to mental health issues such as emotional disorders (Baumeister et al., 2013; Shantall, 2020; Wong, 2012).

**Task orientation**. To lead meaningful lives, humans must have short- and long-term tasks (Frankl, 1985a; 2006; 2014). The tasks must be self-chosen to be meaningful (Fabry, 2013). However, one can freely accept an allocated task. An individual can be sustained during difficult times because of a commitment to a meaningful task (Fabry, 2021). Furthermore, a task can offer a curative value when individuals believe they are the only ones who can accomplish it or perceive themselves as the best person for the task (Fabry, 2021). It is suggested that individuals can truly discover themselves by losing themselves to meaningful tasks (Frankl, 2006; Lukas, 2020).

**Self-transcendence**. Self-transcendence refers to reaching beyond yourself toward others to love or act for a cause that means something to an individual (Fabry, 2021; Frankl, 2006; 2012; Wong, 2016). It represents an individual’s ability to reach above and beyond external conditions towards something or someone other than themselves (Frankl, 2010). It is argued that humans experience meaning in life when they redirect their self-interest to something bigger than and beyond themselves (Fabry, 2021). Studies have shown that pursuing meaningful living may be related to suffering and despair (Baumeister et al., 2013). In other words, individuals may need to courageously and willingly sacrifice personal happiness and well-being to pursue and achieve self-transcendent goals (Wong, 2014).

**Self-distancing**. Self-distancing refers to the ability “to step away from ourselves, look at ourselves from the outside, oppose, and even laugh at ourselves” (Fabry, 2013, p. 137). Frankl argued that self-distancing is important in discovering meaning; it allows individuals to choose their attitudes toward themselves or the burden they carry (Barnes, 2000; Frankl, 2006). Self-distancing thus allows the spiritual person to step away from the psychophysical self. Looking at oneself from the outside enables people to break life patterns and change towards their goals (Barnes, 2000; Fabry, 2013). Self-distancing gives an individual a clearer vision of the areas of freedom or courses of action still available (Lukas, 2020; Shantall, 2020). People can come to understand that problems or symptoms such as feelings of inferiority, fears, emotional outbursts, obsessions, and depression can be modified and possibly overcome through a changed attitude (Fabry, 2021; Frankl, 2006; Lewis, 2019).

Frankl’s theory has been successfully, but rarely, applied in previous psychobiographical studies (Bushkin et al., 2021; Harisunker & du Plessis, 2021; Harry & van Niekerk, 2023; Mayer & Kelley, 2021b). The researchers selected Frankl’s existential theory for this study to illustrate how people can remain optimistic in tragedy. The thesis argues that it is possible to discover or uncover meaning even in the most traumatic, complex, or tragic situations (Fabry, 2021; Frankl, 2006; Lewis, 2019). Studying the lives of individuals who have exhibited courage, resilience, and strength can give hope to others in similar situations that it is possible to live a meaningful life despite the limitations (Mayer et al., 2021a; 2021b; Shantall, 2020; Wong et al., 2022).

### A life sketch of Ellen Johnson-Sirleaf^1^

#### Early life

Johnson-Sirleaf was born on October 29, 1938, in Monrovia, Liberia. After her birth, her parents received a ‘prophecy’ from an old man who said, “This child shall be great. This child is going to lead” (Johnson-Sirleaf, 2010, p. 7). She was the third child in a family of four: Charles, Jennie, and Carney. Her father (Carney) was a Gola (assumed to be the first ethnic group of settlers in Liberia) and her mother (Martha) was a Kru-German (a descendant of a native mother and a German expatriate). Her parents were from poor backgrounds and were fostered (a system where low-income families gave away their children to well-off families so they could go to school and have a better life) into Americo-Liberian families (Cooper, 2017). Carney apprenticed with a practising attorney (due to a lack of law schools in the country) to become a “poor man’s lawyer” (p. 11). He was also the first indigenous person to be elected to the Liberian House of Representatives. By local standards, the family was regarded to be in the upper middle class (Scully, 2016). Johnson-Sirleaf recalls a happy childhood because of her father’s status. Her mother was an elementary school owner, teacher, and Presbyterian preacher.

Johnson-Sirleaf attended a prestigious preparatory school named the College of West Africa between 1948 and 1955. At school, she was bullied because of her light skin, resulting in other children calling her a red pumpkin. At about nine years old, she had her wrist cut repeatedly with a razor to receive a *fighting potion* from her grandmother in preparation for a fistfight with another girl (Berger, 2010). In 1956, when Ellen was 18 years old, the family’s fortunes turned, as Carney suffered a stroke that paralysed his right side, leading to his demise in 1957. Subsequently, the family income plummeted. Consequently, Martha shouldered the burden of caring for and providing for the family.

#### Marriage

The family’s change in fortunes temporarily put Johnson-Sirleaf’s college prospects on hold. She mentions that she had to give in to tradition as she saw no other options besides getting married (Cooper, 2017; Scully, 2016). In 1956 she married James (Doc) Sirleaf (who was seven years older than her). She had four sons in quick succession between 1957 and 1961 and became a housewife or homemaker (Bourlin, 2013; Cooper, 2017). For many Liberian women, the rest of their lives would have evolved around raising children, nurturing the family, and working in menial employment (Cooper, 2017; Scully, 2016). But that was not the life she wanted for herself.

However, before she could pursue her path, she was a victim of domestic abuse, from verbal to physical abuse, at the hands of Doc (Cooper, 2017; Scully, 2016). The abuse happened at a time when women did not file charges against their husbands but only reported it to their families. As she saw her friends moving abroad to pursue tertiary education, she felt ‘trapped’ with an abusive husband, four young sons, and no future in sight (p. 7). Despite feelings of entrapment and domestic abuse, she travelled with her husband to the United States of America (USA), where she earned an accounting degree from the Madison Business College in Wisconsin. However, this came at a cost, as she had little time to care for her children in her quest for education and her ambitions, especially her youngest son (Al Jazeera English, 2010). Unfortunately, the domestic abuse continued even when she was in the USA. Despite the abuse, Johnson-Sirleaf completed her studies. In 1961, upon her return to Liberia, she initiated the divorce process as she had suffered domestic abuse from the start of her marriage. By filing for divorce, she was at risk of further abuse if her ex-husband refused to accept the divorce, as the law favoured men (Cooper, 2017; Wellesley College, 2019). It was a common tradition that after divorce, the children remained with the father regardless of the circumstances leading to the divorce. Consequently, she was again separated from her sons, and that caused her “intense” pain (p. 41).

#### Political career

After her divorce, she returned to the U.S. to continue her education, which she highlighted as an escape from her ex-husband (Cooper, 2017; Scully, 2016). She acquired a master’s degree in public administration from Harvard University in 1971. This contributed to her rapid ascendency in the Liberian government, initially serving as an Assistant to the Minister of Finance. In 1979, Johnson-Sirleaf was appointed the first female Minister of Finance in Liberia under President William Tolbert. Although she was part of the government, she spoke against engagement in corruption by government officials.

In 1980, President Tolbert was removed and executed in a coup d’état led by General Samuel Doe. The coup d’état also resulted in the execution of 13 of Johnson-Sirleaf’s former colleagues (Berger, 2010). As a survivor of the executions, she was determined to ensure that her colleagues did not die in vain (Al Jazeera English, 2010). Despite the executions, her family being in exile, the suspension of the constitution, and the establishment of military rule, she stayed and served in President Doe’s government as the president of the Liberian Bank for Development and Investment (Cooper, 2017; Scully, 2016). She continued speaking out against corruption, which angered President Doe, and she had to escape the country with the help of the World Bank in 1980.

In 1984, she returned to Liberia as she felt compelled to serve her country. Again, she gave another speech against corruption, resulting in her house arrest and imprisonment for sedition (Berger, 2010). Following significant international pressure, the government subsequently dropped the charges. Instead of moving overseas, in 1985 she contested and won the elections for a senatorial seat. After the elections, Johnson-Sirlead declined to assume the senatorial seat, citing that the military government rigged the polls. She had to endure verbal abuse, death anxiety, imprisonment, uncertainty, and fear because of her defiance. President Doe had Johnson-Sirleaf re-arrested on charges of sedition, leading to her imprisonment for seven months. In 1986, upon being released from prison, she went into exile to escape the increasingly oppressive military government. During that period, Johnson-Sirleaf held several positions for organisations such as the HSBC Equator Bank, the United Nations, and the World Bank. Even in exile, when the organisations she worked for did not allow her to be involved in politics, she remained invested in Liberian politics.

She was one of the supporters of Charles Taylor and hoped for better living conditions and the ending of the civil war. However, the civil war continued until 1996 as Taylor further inflicted more suffering on the Liberians. In 1997, Johnson-Sirleaf resigned from her post as an Assistant Secretary-General of the U.N. to contest the Liberian presidential elections. Unfortunately, she came second in the polls. Taylor invited Johnson-Sirleaf to join the government after the election. Even U.S. President Carter is said to have told her to serve in Taylor’s government, an opportunity she turned down. Taylor is believed to have silenced his opponents through assassinations and indicting them of treason (Scully, 2016). In 1997, Taylor accused and charged Johnson-Sirleaf with treason and pardoned her in 2001.

Consequently, she remained in self-imposed exile in Cote d’Ivoire (Ivory Coast). In the early 2000s, Johnson-Sirleaf was a key participant and influencer of gender equality and the women's movement. She co-authored a document with Elizabeth Rehn (Rehn et al., 2002), which provided a framework for discussions and policymaking about the inclusion of women in peacebuilding. In April 2001, despite her vocal stance against corruption, reports implicated her in corruptive dealings. For instance, she was named as a director of an offshore bank with no legitimate Liberia and faced accusations of corruption within her political party and nepotism (Herbert, 2014).

In August 2003, Johnson-Sirleaf returned to Liberia following the ousting of Taylor from the country. She contested the presidential elections in 2005 and won the elections, making her the first-ever elected female head of state in Africa. At the inauguration, Johnson-Sirleaf spoke of economic development, ending corruption, and the need for good governance, reconciliation, and responsibility (Scully, 2016). Johnson-Sirleaf stated that her inner strength and desire to succeed helped her succeed (Al Jazeera English, 2010). Facing and surviving difficult circumstances made her more ambitious and courageous, and she had to make unimaginable sacrifices, for example, leaving her children (Wellesley College, 2019). Johnson-Sirleaf noted that recalling the older man's prophecy made her continuously return to Liberia as she wanted to challenge the status quo (Wellesley College, 2019).

## Methodology

### Research design

Psychobiographical research entails the application of psychological theory and biographical information to construct the lived experiences of extraordinary individuals and illuminate patterns in behaviour, thinking and feelings (Ferrer & Ponterotto, 2020; Sollmann & Mayer, 2021; Mayer et al., 2020; Ponterotto, 2018; Prenter et al. 2019; Van Niekerk et al., 2019). However, psychobiographical studies about women in politics and women leaders in African contexts are limited (Mayer, 2022; Mayer et al., 2022; Panelatti et al., 2021). This study employs a qualitative, longitudinal, singlecase, psychobiographical design to describe and explore a selected period of Johnson-Sirleaf’s life (Ferrer & Ponterotto, 2020; Schultz, 2005). The method can also be described as morphogenic (focusing on an individual within a holistic context and studying an individual as a holistic unit) (Burnell, 2013; Elms, 1994; Mayer, 2017).

### Sampling

Johnson-Sirleaf was selected for this study as she is an extraordinary individual who was courageous, brave, and regarded as an influential contemporary political leader who flourished in a male-dominated society (Steady, 2011; Wilson, 2015). She was purposively chosen for her contributions in Liberia towards democratic rule, women empowerment, and economic and social development (ACET, 2022). Johnson-Sirleaf navigated a complex political landscape to be the first elected female president in Africa and led Liberia through reconciliation and healing from a decade of civil war and the Ebola crisis (Cook, 2018; Dennis, 2006).

### Data collection and analysis

The researchers used publicly available material as data sources (Ponterotto, 2017a, 2017b). Primary and secondary sources included an autobiography (Johnson-Sirleaf, 2010), interviews (Al Jazeera English, 2010; Wellesley College, 2019), biographies (Cooper, 2017; Scully, 2016), and internet sources (ACET, 2022; Berger, 2010; Bourlin, 2013; Wilson, 2015). Relevant themes or units were filtered out using Yin’s strategy (Yin, 2013). Frankl’s tri-dimensional ontology, particularly human spirit resources, was used to identify themes in the collected data (deductive thematic analysis), and the study’s aims guided the data analysis (Yin, 2013; Fouché et al., 2017).

### Ethical considerations

Ethical considerations are essential in psychobiography, especially when researching living subjects and their private lives. To this effect, we sent a consent form to the Ellen Johnson Sirleaf Presidential Centre for Women and Development; however, no response was received. Nonetheless, we ensured there was no potential for the participants and their relatives to be embarrassed or harmed during the presentation (Ponterotto, 2017a,b; Ponterotto & Reynolds, 2017). We were also respectful and empathetic to the individual under study by being responsible with the data and findings and only using information available in the public domain (Elms, 1994; Ketefian, 2015; Ponterotto, 2015; 2017a, 2017b).

## Findings and Discussion

The findings of Johnson-Sirleaf’s journey were discussed using the resources of the noetic dimension. Frankl recognised that the spirit and psyche are both significant in human behaviour and have observable signs. However, the two have differences; in the psyche, humans are driven by needs, emotions, and instincts, while in the spirit (the area of human freedom and responsibility) humans are the drivers (they are free to make choices) (Lukas, 2020). Fabry (2013) suggested that one must be aware of the spiritual treasure chest and use the contents within to lead a fulfilled (meaningful) life. Specific examples of how Johnson-Sirleaf utilised the medicine chest of logotherapy to discover meaning are highlighted in this section. The selected five themes from Frankl’s theory, i.e., conscience, will to meaning, task orientation, self-transcendence, and self-distancing, evident in Johnson-Sirleaf’s existential journey, are presented in this section. The broad themes are described and interpreted within the socio-historical context of the subject.

### Resources of the human spirit

#### Conscience

One of the most prevalent themes was the utilisation of conscience. From childhood, Johnson-Sirleaf appears to have openly opposed patriarchal values (Cooper, 2017). She engaged in activities mainly for boys, for instance, playing football and taking up leadership roles in school (Johnson-Sirleaf, 2010). Erikson (1968) argued that the period from 12 to 20 years is essential for identity formation. It is a period characterised by exploring beliefs, values, and goals while searching for personal identity and a sense of self (Erikson, 1968; 1970). During this time, however, her safe and secure environment was abruptly disrupted by her father’s illness and subsequent death.

Consequently, Johnson-Sirleaf temporarily abandoned her ambitions by marrying at 17 and giving birth to four children in five years. During this time, she conformed to the prevailing societal norms and values. However, she felt isolated and inadequate as she was not achieving like her former schoolmates, which gave her the drive to do something about her life: “I felt that something was missing, that this was not quite the life I was meant to have” (Johnson-Sirleaf, 2010, p. 32). Studies have shown that individuals’ search for meaning and identity intensifies in the emerging adulthood phase (Erikson, 1968; 1970; Lukas, 2019). In Johnson-Sirleaf’s life, this period seems to have been frequented with questions of her identity, future, and the meaning and purpose of her life.

Furthermore, in a male-dominated society, she was expected to endure domestic abuse inflicted by her ex-husband. Nevertheless, it seems that she discovered *the meaning of the moment*, responding to opportunities for meaning in the moment to lead a meaningful life, through her decisions that were guided by her conscience (Fabry, 2013; Frankl, 1985a, 1985b; Lewis, 2019). An individual can discover the meaning of the moment by choosing between traditional values and individual conscience (Barnes, 2000; Fabry, 2021; Frankl, 2006; 2012; 2014; Shantall, 2020). In Johnson-Sirleaf’s case, she often faced the contradiction between traditional values and individual conscience. For example, choosing between her children/being a stay-home mother and pursuing education overseas; the value of making a career as a woman and the value of being a home marker (*good Liberian wifehood*); choosing between remaining in an abusive marriage and the prospect of losing her children; the value of a well-paying job and the value of following one's calling or purpose (Cooper, 2017; Johnson-Sirleaf, 2010; Scully, 2016). This may suggest that she pursued the unique meaning of the different situations and found personal meanings rather than relying on traditional values (Barnes, 2000; Fabry, 2013; 2021). She had to reconstruct her beliefs and values to form a new identity, shaped partly by her upbringing, pain and suffering. Johnson had to find meaning within her new identity of being a divorcee and single parent.

Johnson-Sirleaf also courageously criticised and openly opposed the military governments for failing to run the country (Cooper, 2017; Scully, 2016). She faced the prospect of death, imprisonment in military prisons, and threats, yet remained defiant. For her, it was meaningful to encounter and heroically accept the fate that awaited her for defying the government (Fabry, 2013; 2021; Frankl, 2006; 2014). It appears that she believed in her freedom to choose what she wanted to do and took responsibility for her choices (Frankl, 2006, 2014; Lukas, 2020). Moreover, it seems she was constantly guided by her conscience even when it conflicted with the established values (Fabry, 2021). Johnson-Sirleaf's life shows us that conscience permits us, under certain circumstances, to take a stand against impulses, physical limitations, traditions, customs, laws, drives, and environmental influences (Fabry, 2013; Lewis, 2019). Our profoundly rooted conscience enables us to find the specific meanings of the moment of our individual life.

#### Will to meaning

Johnson-Sirleaf's will to meaning is the second theme from the findings. She had various opportunities to attain pleasure or power in the country during the rule of the warlords. She was offered positions in General Doe’s and Charles Taylor's governments (Johnson-Sirleaf, 2010; Scully, 2016). Frankl argues that power is not an end but a means to pursue meaning (Barnes, 2000; Frankl, 1985a, b; 2006; 2014). Notably, despite threats, psychological torture, and imprisonment, Johnson-Sirleaf rejected the power as it would not have been used meaningfully. She wrote: “If I wanted to be successful according to the terms of success that had long dominated our society, was to go with the flow. But I could not do that” (Johnson-Sirleaf, 2010, p. 67).

Another instance which shows her will to meaning is in her career choices. She held several positions in prestigious organisations (e.g., the U.N. and World Bank). She could have enjoyed a luxurious life away from her country’s oppression and civil war in such posts. However, she still decided to relocate to Liberia and, in 1997 ran for the presidency against a warlord, Charles Taylor (Johnson-Sirleaf, 2010). This may suggest that she sought a more meaningful career than a well-paying, meaningless profession (Barnes, 2000; Fabry, 2021; Frankl, 2012). She found meaning in pursuing a career that she regarded as meaningful. Johnson-Sirleaf seemed to have served others with faith and demonstrated selfless leadership for the greater good (Wong et al., 2022).

#### Task orientation

The third theme, task orientation, looks at the tasks that Johnson-Sirleaf completed. Johnson-Sirleaf had short-term tasks such as gaining corporate world experience and challenging the warlords and status quo (including the ideology of patriarchy) (Johnson-Sirleaf, 2010). Her long-term tasks included empowering marginalised people (women and children). As discussed previously, there were times when she was forced to take on tasks that were not aligned with her values. However, she did not accept the tasks as they would not have fulfilled her, as they contradicted her beliefs and were not self-chosen (Fabry, 2021; Marshall & Marshall, 2012). Although she faced the threat of imprisonment and death, she refused to take up the senatorial position in the military government as she did not prescribe to the values of the government. She was again arrested and taken to the army’s ragtag military post, which was exceedingly dangerous for a prisoner (Johnson-Sirleaf, 2010). Other prisoners cried and pleaded for their lives before they were shot to death (Johnson-Sirleaf, 2010). But she did not plead for her life or beg for mercy from her oppressors. The belief that she had a meaningful task that awaited her likely allowed her to endure her unnecessary suffering (Fabry, 2021; Frankl, 2006). She stated, “I had always looked for challenges, and this (leading a political alliance in Liberia) was one of the greatest of all” (Johnson-Sirleaf, 2010, p. 211).

#### Self-transcendence

Self-transcendence is another prominent theme that surfaced during the analysis. Johnson-Sirleaf was able to go beyond her suffering and committed to the cause of freeing Liberian citizens and empowering women and children. Another example of her finding meaning by going beyond herself was when she embraced a female prisoner who had been a victim of sexual assault (Johnson-Sirleaf, 2010). Although facing the same fate, Johnson-Sirleaf was able to offer comfort to this prisoner. Furthermore, she had an extraordinary career away from the civil war, and her family members were safe in other countries. However, despite these factors, she remained invested in the liberation of her country. In another instance of transcendence, after being released from prison, Johnson-Sirleaf had the chance to leave and start a new life in a war-free country. However, she remained in the country despite warnings from General Doe about her continued involvement in the country’s politics and elections. She suffered humiliation and indignity for refusing to take the senatorial seat she had won in the polls.

Moreover, as a religious person raised in a religious family, she strengthened herself by saying, “Be still and know that I am God” (Johnson-Sirleaf, 2010, p. 130). Because of her faith, she was unafraid of what would happen during the court trial and imprisonment. Frankl (2012) argued that meaning is seen on two levels: life’s *ultimate meaning* and the *meaning of the moment*. Ultimate meaning involves the belief that there is an order in the universe in which individuals have a part (Fabry, 2013). The order can be formulated in religious or secular terms, life, nature, or the ecosystem. Johnson-Sirleaf found meaning in believing in God’s will and word, although she did not know her fate. She found meaning in her faith and the support from other people, especially women. Being religious, it also seems she believed in the prophecy her parents received about her being great and a leader. It also appears that the prophecy acted as her inner motivation for some of the decisions she made. Her transcendental perspective on life helped her endure the suffering, death anxiety and fear the warlords caused (Fabry, 2021; Frankl, 2012; 2014).

Looking at Johnson-Sirleaf's life, there is evidence that she was able to look beyond the personal constraints that she faced to discover the meaning of the situation and seek out her calling or purpose to serve the greater good (Wong, 2016). Lastly, she could go beyond the limitations of the psychophysical and psychosocial dimensions into the spiritual dimension, where she could discover the meaning of her life (Lukas, 2020; Shantall, 2020). It also shows how humans are motivated by their will to meaning – they tend to reach out for activities or tasks and experiences that are meaningful to them. If a person is determined and committed to a self- chosen goal or mission, they will be prepared to forego pleasures and disregard drives, as Johnson-Sirleaf did (Barnes, 2000; Fabry, 2013; 2021; Frankl, 1985a, b). Interestingly, although she loved her children, she neglected them in search of personal identity and a sense of self: “One thing I had always believed in was my potential, and I knew it did not lie in simply raising them (her children)” (Johnson-Sirleaf, 2009, p. 33). Although her children were important, her will to meaning drove her to search for her unique life meaning, showing that people draw different meanings from similar situations.

#### Self-distancing

Self-distancing is the final theme used to analyse the findings. Johnson-Sirleaf was exposed to potentially traumatic and stressful events in her life, such as freezing in front of a congregation as an 8-year-old, being bullied for her light skin (being called a red pumpkin), domestic violence, imprisonment, civil war, witnessing the effects of genocide, exposure to death and threatened death, and the violent execution of her former colleagues (Cooper, 2017; Johnson-Sirleaf, 2010; Scully, 2016). Furthermore, each time she was arrested, and she was arrested on a few occasions, she carried the anxiety of an unknown fate (Johnson-Sirleaf, 2010). In the 2005 presidential elections, she also faced many negatives, including being too light-skinned, too mature, having political baggage, being too educated, and society insisting on male leadership (Johnson-Sirleaf, 2010). In what Frankl (2014) referred to as the defiant power of the human spirit, Johnson-Sirleaf took a stand against these adverse circumstances (external and psychophysical factors) in her spiritual dimension through her chosen attitude (Marshall & Marshall, 2012). She was able to choose how she responded to the situation and herself in spite of the limitations. Self-distancing allowed her to view the problems as something she had, not something she was (Barnes, 2000).

The following quote summarises Johnson-Sirleaf's commitment and engagement with life:

“Sometimes people find this surprising about me, but I believe that perhaps the thing that has sustained me during my many close calls is that I have a certain frame of mind that banishes fear. I suppose that is the part of me that believes in predestination” (Johnson-Sirleaf, 2010, p. 223).

It is apparent that despite the hindrances, tragedies, limitations, and traumas, her inner motivation to discover, uncover, maintain, and embrace meaning remained present throughout the period under study. We thus argue that, through her responses to life situations, she was able to discover and find meaning.

## Conclusion and Recommendations

Johnson-Sirleaf's life illustrates how humans are free agents who are not always free from psychophysical or psychosocial conditions but can take a stand within and against those conditions (Frankl, 1985a, 1985b). Frankl does not disregard our physical and psychological conditions, drives, or the importance of childhood experiences, environment, and upbringing (Barnes, 2000; Frankl, 2006; 2012; 2014; Shantall, 2020). He argues that even under the most restrictive and miserable conditions or circumstances, we have an area of freedom in which we can determine our actions, experiences, or, at the very least, our attitudes towards those conditions (Fabry, 2021). Human personality is thus not only shaped by genes or environment but by what we do with our genetic make-up, how we deal with the various circumstances we face and how we relate to the others that cross our paths.

The freedom to choose is found within the noetic dimension. Accessing this dimension allows humans to go beyond themselves and discover essential meanings and values of their existence. Including the noetic dimension in studying human development does not mean we deny the existence of emotions and drives (pleasure) and the desire to be prosperous and materially secure (power) seeking expression in behaviour. Including this dimension means acknowledging that humans can choose whether to be driven by or resist those impulses. The noetic dimension tells us that humans can deny themselves pleasure or power if they see a meaning behind their rejection. Therefore, a human being is not only a result of heredity or environment but also of their choices.

Johnson-Sirleaf's life is a typical example of humans operating within the noetic dimension. Despite the existence of psychosocial and psychophysical factors, she could access the resources of the spiritual dimension, which allowed her to live a meaningful life. Johnson-Sirleaf’s life contributes significantly to the search for meaning narratives and empowers and inspires previously disempowered groups. Finding meaning in adverse circumstances helps make life more bearable. Suffering is shaped not only by pain, loss, meaninglessness, hopelessness, and questioning, but also by growth, purposiveness, and courage. However, it is also important to note that one does not have to suffer to discover the meaning of their life.

In conclusion, this study described and explored the search for and discovery of meaning in Johnson-Sirleaf's life despite the many tragedies and life-threatening circumstances she faced, using Frankl’s concept of dimensional ontology, specifically the noetic dimension. The study focused on a specific period when she faced many tragedies, such as being bullied at school, her parents’ death, being a domestic abuse victim, witnessing genocide, and imprisonment. Johnson-Sirleaf's life illustrates the idea that psychophysical or psychosocial factors do not entirely determine the behaviour of humans. We can take a stand against all causes that may condition us in the noetic dimension. To a greater extent, humans are the masters of their lives as they are more than mere victims of their past or learnings.

Nonetheless, we note that the conclusions of this study are limited to the analysis of Johnson-Sirleaf's life within the scope of a selected theoretical and methodological standpoint as well as to a specified period of her life. Hence, further insights into Johnson-Sirleaf’s development could be obtained by applying alternative theoretical frameworks. Despite these limitations, this study highlights that Frankl’s theory can be helpful in comprehending humans in their totality and how they discover meaning within tragedy.

## Footnotes

1 The information in this section is gleaned from Johnson-Sirleaf's autobiography: *This child will be great: Memoir of a remarkable life by Africa's first woman president* (Johnson-Sirleaf, 2010), unless stated otherwise.

## Action editor

Lars-Gunnar Lundh served as action editor for this article.

## Open access

This article is distributed under the terms of the Creative Commons Attribution 4.0 International License (http://creativecommons.org/licenses/by/4.0/), which permits unrestricted use, distribution, and reproduction in any medium, provided you give appropriate credit to the original author(s) and the source, provide a link to the Creative Commons license, and indicate if changes were made.
